# Diphencyprone Induced Vitiligo: A Case Report

**DOI:** 10.1155/2012/356236

**Published:** 2012-07-01

**Authors:** Mohammad Ali Nilforoushzadeh, Gelavizh Keshtmand, Fariba Jaffary, Abbas Kheirkhah

**Affiliations:** ^1^Skin and Stem cell Research Center, Tehran University of Medical Sciences, Tehran 19377957511, Iran; ^2^Center for Research and Training in Skin Disease and Leprosy, Tehran University of Medical Sciences, Tehran 1416613675, Iran; ^3^Cancer Research Center, Cancer Institute of Iran, Tehran University of Medical Sciences, Tehran 13145-258, Iran; ^4^Skin Diseases and Leishmaniasis Research Center, Isfahan University of Medical Sciences, Isfahan 81876-98191, Iran

## Abstract

Diphencyprone (DCP) is a contact sensitizer which is used to treat dermatological disorders with an immunological origin, such as extensive alopecia areata (AA). Vitiligo is a rare but known side effect of DPCP therapy which is formed in the treatment site or remote areas. In this paper a 37-year-old man developed alopecia totalis with loss of eyebrows and eyelashes who presented some vitiligo patches on his scalp and arm distant from the location of DPCP application and a 42-year-old woman with 25 years history of hair loss and 3 months DPCP application who revealed some vitiligo patches on the scalp with distant to the site of application at the 6th week are reported. Considering the absence of personal and family history of Vitiligo in our two cases, the hypothesis of latent Vitiligo is not proved. The positive patch test in left arm of one of the patients also suggests the direct role of DPCP as the cause of Vitiligo occurrence. As the development of vitiligo by DCP is unpredictable and the depigmentation may persist indefinitely, it is important to inform all patients about this potential adverse effect before starting the treatment.

## 1. Introduction

Alopecia areata (AA) is one of the most frequently organ-restricted diseases, characterized by non-scarring hair loss, considered to be an autoimmune disorder [1] and approximately occurs in 2% of the population [2]. The management varies widely among dermatologists. Treatment modalities include corticosteroids, topical irritants, photochemotherapy (PUVA), contact immunotherapy, and biological drugs [3]. No treatment has been shown to alter the course of the disease or to have a significant long-term benefit [4]. Contact sensitizers include dinitrochlorobenzene (DNCB), diphenyl cyclopropenone (DPCP), and squaric acid dibutyl ester (SADBE). Vitiligo is one of the undesirable and rare adverse effects of topical sensitizers [5]. This complication has become a principal challenge to the dermatologists due to its Resistance to treatment [5]. We used DPCP for the mangement of adults with more than 50% scalp involvement [6]. At present, topical immunotherapy with DPCP is considered the most effective treatment of AA with success rates ranging from 4% to 85% [7]. In this paper, two cases of AA who developed vitiligo after treatment with DCPC are introduced.

## 2. Main Text

### 2.1. Case 1

**Figure 1:**
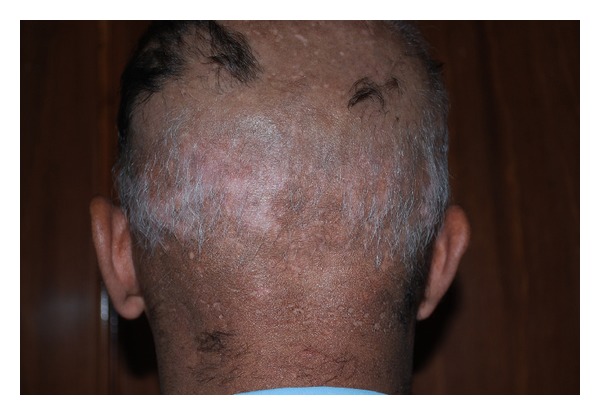
Vitiligo patches on scalp with white hair.

**Figure 2:**
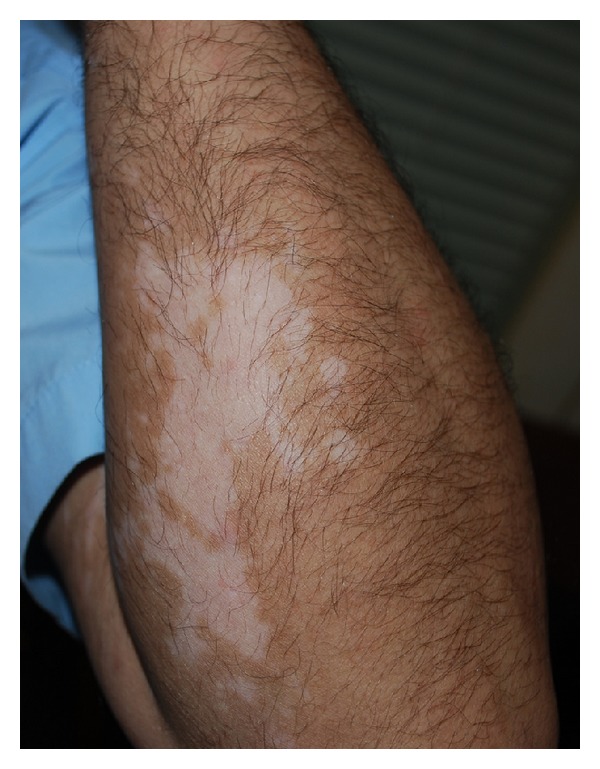
Vitiligo patches on left arm distant from the location of DPCP application.

A 37-year-old man developed alopecia totalis, loss of eyebrows and eyelashes and widespread thinning of the hair since 31-years-old with no personal or family history of vitiligo. He had been treated with corticosteroid with diagnosis of AA with good response to the treatment but due to adverse effects, the treatment was discontinued and DPCP 0.5% for 4 months (once a week) in hair loss regions was commenced following washing the areas 5 hours later with no prior sensitization that resulted in the first sign of hair regrowth in early fourth week and nearly total hair of head with regrowth of eyebrows, eyelashes and beard at the third month. Some vitiligo patches were revealed on his scalp and arm distant from the location of DPCP application and they preceded by contact dermatitis of the scalp (Figures 1 and 2). Then dark hair in the depigmented patch was gradually replaced on the scalp with white hair. Complete blood count and biochemical profile were normal and antinuclear antibody test was negative but antithyroid peroxidase was in high range (29.7; normal ranges up to 16) in this case.

### 2.2. Case 2

A 42-year-old woman with 25 years history of hair loss and a mean duration of 3 months DPCP therapy before the onset of vitiligo is the second case. The first presentation of the disease was generalized hair loss. She had been treated with corticosteroid with the diagnosis of AA for several years but due to low effectiveness, the treatment failed. She complained of coin shape hair loss in her scalp and body since 17-years-old. In physical examination rounded irregular patches varying from 1 to 2 cm mostly in occipital and parietal regions were observed. She had no personal or familial history of vitiligo. In our center, the patient was treated with intralesional corticosteroid and topical minoxidil but due to the remain of hair loss patches, topical DPCP 0.5% was prescribed once a week without prior sensitization. The treatment resulted in the first sign of white hair regrowth at the 6th week, and some vitiligo patches appeared on the scalp with even white and dark hair, as well as on her face, after 5 months distant to the site of application. A marked reaction with macular erythema was obvious on her scalp after the first time application of DPCP but it subsided a few days later. There has been no relapse of alopecia in several months followup after discontinuing of DCP therapy and the depigmented areas remained unchanged. 

A growth of dark hair within the vitiliginous patch on both patients was probably due to activation of follicular melanocytes by a nonspecific effect of contact dermatitis. Vitiliginous patches at the sites of contact with DPCP may be the consequence of postinflammatory hypopigmentation.

## 3. Discussion

Alopecia areata (AA) is a disease of unknown cause. Evidence of an autoimmune etiology of this disease is still lacking [8]. T-lymphocytic infiltrate around and inside the bulb of hair follicles is reported in patients with untreated AA [9]. Genetic factors such as HLA typing, atopic syndrome, thyroid antibodies, and autoimmune diseases have been associated with subtypes of AA [3]. Although the mechanism of action of DPCP has not been clearly defined, it is considered to be one of the effective modalities for the treatment of AA through induction of an allergic contact dermatitis [8]. It has been proposed that different T cells migrate to the treated area which increase the clearance of the follicular antigen [3] predicting 100% response rate for patients with 26% to 49% hair loss, 88% for patients with 50% to 74% hair loss, 60.3% for patients with 75 to 99% hair los,s and 17.4% for patients with AA totalis/universalis [9]. Although the treatment of patients suffering from AA with DPCP presents high response rates similar to those reported by previous studies, the potential risk of vitiligo must be considered. The treatment is adequately tolerated by most of the patients, and they are willing to repeat the treatment courses in case of relapse. As the development of vitiligo by DCP is unpredictable and the depigmentation may persist indefinitely, it is important to inform all patients about this potential adverse effect before starting the treatment [10]. In this paper the considerable point was complete recovery of the AA in both patients a few months later but both patients developed an eczematous reaction at the application site and revealing the white hair (Leucoderma) and vitiligous patches was the main cause of discontinuing therapy. The treatment was interrupted and after 3 months, the hypopigmented lesions did not develop pigmentation. The well-documented high relapse rate in medical literature was also observed in our patients after stopping the treatment [3]. The patch test was performed with DPCP 0.5% in the left arm of the male patient, and an erythematous reaction was observed after a few days. The hypopigmented lesion at the patch test site appeared after three weeks. Treatment of Leucoderma includes discontinuation of DPCP, application of topical steroids or phototherapy. In most cases, repigmentation may occur with treatment, but complete recovery is uncommon [11]. In this paper the patients were asked to refer for phototherapy for the vitiligo lesions but they lost to followup. Henderson and Ilchyshyn [11] reported a case of extensive hair loss who was under the treatment of 2% DCP solution that resulted in patchy spontaneous regrowth. After this treatment, extensive dermatitis on the scalp and subsequently on the hands and legs and low-grade dermatitis were developed followed by extensive depigmentation on the right half of the scalp and on the face and neck thirteen weeks later.

Both Vitiligo and AA are based on autoimmune origin and sometimes are reported in association with each other while concurrency of both diseases at the same time is very rare [12]. Yadav et al., Adams et al., and Dhar et al. have reported some cases of concurrency of both diseases [12–14]. The hypopigmented areas may be the primary course of the disease which is revealed as the result of Koebner phenomenon in suspicious patients to Vitiligo or they may be the consequence of direct cytotoxic effect of treatment with DCPC on Melanocytes following systemic absorption (particularly in distant areas) that the distinction of these two is not possible [5, 15]. Considering the above-mentioned statements about the association of AA and Vitiligo in the absence of treatment with DCPC, it can be assumed that DCP application results in revealing latent Vitiligo in suspicious patients but regarding the absence of personal and family history of Vitiligo in our two cases, the mentioned hypothesis is not proved [5, 15]. The performed patch test in the first case which resulted in erythematous reaction following hypopigmented patch in the test region in left arm can demonstrate the consequence of secondary Vitiligo and direct role of treatment with DCPC that is similarly shown in Mario Cezar et al.'s report [5, 15].

Our patient did not recover the pigmentation after topical corticosteroid therapy for vitiligo; however, we require further evaluations of the hypopigmented patches and hair in long-term and also phototherapy for the treatment.
